# Iron oxide/hydroxide–nitrogen doped graphene-like visible-light active photocatalytic layers for antibiotics removal from wastewater

**DOI:** 10.1038/s41598-023-29927-9

**Published:** 2023-02-15

**Authors:** R. Ivan, C. Popescu, V. A. Antohe, S. Antohe, C. Negrila, C. Logofatu, A. Pérez del Pino, E. György

**Affiliations:** 1 National Institute for Lasers, Plasma and Radiation Physics, PO Box MG 36, 077125 Măgurele, Ilfov Romania; 2 Faculty of Physics, University of Bucharest, Atomiștilor 405, 077125 Măgurele, Ilfov Romania; 3 Institute of Condensed Matter and Nanosciences (IMCN), Université Catholique de Louvain (UCLouvain), Place Croix du Sud 1, 1348 Louvain-La-Neuve, Belgium; 4 Academy of Romanian Scientists (AOSR), Splaiul Independenței 54, 050094 Bucharest, Romania; 5 National Institute for Materials Physics, PO Box MG 7, 077125 Măgurele, Ilfov, Romania; 6 Instituto de Ciencia de Materiales de Barcelona, Consejo Superior de Investigaciones Científicas (ICMAB-CSIC), Campus UAB, 08193 Bellaterra, Barcelona Spain

**Keywords:** Pollution remediation, Photocatalysis

## Abstract

Hybrid layers consisting of Fe oxide, Fe hydroxide, and nitrogen doped graphene-like platelets have been synthesized by an eco-friendly laser-based method for photocatalytic applications. The complex composite layers show high photodecomposition efficiency towards degradation of antibiotic molecules under visible light irradiation. The photodecomposition efficiency was investigated as a function of relative concentrations of base materials, Fe oxide nanoparticles and graphene oxide platelets used for the preparation of target dispersions submitted to laser irradiation. Although reference pure Fe oxide/Fe hydroxide layers have high absorption in the visible spectral region, their photodecomposition efficiency is negligible under the same irradiation conditions. The high photocatalytic decomposition efficiency of the nanohybrid layer, up to 80% of the initial antibiotic molecules was assigned to synergistic effects between the constituent materials, efficient separation of the electron–hole pairs generated by visible light irradiation on the surface of Fe oxide and Fe hydroxide nanoparticles, in the presence of conducting graphene-like platelets. Nitrogen doped graphene-like platelets contribute also to the generation of electron–hole pairs under visible light irradiation, as demonstrated by the photocatalytic activity of pure, reference nitrogen doped graphene-like layers. The results also showed that adsorption processes do not contribute significantly to the removal of antibiotic molecules from the test solutions. The decrease of the antibiotic concentration under visible light irradiation was assigned primarily to photocatalytic decomposition mechanisms.

## Introduction

Contamination of water resources results in uncontrolled exposure of human beings as well as flora and fauna to hazardous and hardly-degradable organic compounds, industrial byproducts, oil pollution, agricultural and pharmaceutical wastes^1,2^. Among organic pollutants, pharmaceutical wastes, and in particular antibiotics, are generating an increasing global concern, their massive presence in the aquatic environment representing an extremely high risk to the natural ecosystem. Due to numerous side effects, such as bone marrow suppression leading to aplastic anemia and leukemia, as well as neurotoxic and allergic reactions, continued exposure to antibiotics endanger severely human health^3,4^. The development of bacterial drug resistance is another consequence of the high amount of antibiotics released in the aquatic environment^3^. Chloramphenicol sodium succinate (CAP, C_15_H_15_C_l2_N_2_ Na_2_O_8_) is a broad-spectrum antibiotic, able to impede the growth of both Gram-positive and Gram-negative bacteria^5^. However, as other pharmaceutical compounds, CAP molecules can be only partially removed from aquatic resources through conventional treatment processes, generating also byproducts^6^. Therefore, the development of additional methods for a more efficient removal of recalcitrant organic contaminants from the aquatic environment is of paramount importance.

Solar light induced photocatalytic degradation of hazardous organic contaminants based on advanced oxidation processes (AOP) is considered to be a promising, non-toxic, cost effective, and environmentally friendly alternative to conventional water treatment processes^7^. Through the AOP reactions between the photogenerated electrons and holes as well as oxygen and water molecules taking place near the surface of semiconductor materials, several reactive oxygen species (ROS) are generated which induce the decomposition of recalcitrant organic molecules. Superoxide anion radicals (^**·**^O_2_^−^), hydrogen peroxide (H_2_O_2_), singlet molecular oxygen (^1^O_2_*), and hydroxyl radicals (^**·**^OH) are the major ROS and main intermediates in photocatalytic degradation processes^8^. Among transition metal oxide semiconductor photocatalyst, TiO_2_ has attracted the greatest interest due to its chemical and photochemical inertness, low cost, non-toxicity, and photoinduced superhydrophilicity^9^. However, drawbacks as wide band gap limiting the absorption region to the UV spectral range and reduced lifetime of the photogenerated electron–hole pairs restrict its technological application^9,10^. Various pathways were proposed to overcome these inconveniences. Addition of CdS quantum dots or water soluble carbon quantum dots were reported to increase photocatalytic activity of TiO_2_ nanofibres under UV light irradiation, due to efficient charge separation^11,12^. Au@Ag core–shell particles contribute to absorption of UV and visible radiations, the generated electrons being transferred to the conduction band of TiO_2_ nanofibres. The charge separation between Au@Ag core–shell particles and TiO_2_ nanofibres prevents recombination of electron and hole pairs, leading to enhanced photocatalytic efficiency^13^. Visible light absorption of TiO_2_ was also enhanced by inclusion of anion or transition metal dopants^14,15^.

As an alternative, lower band gap metal oxides, as Fe oxides and hydroxides are currently considered for photocatalytic applications, owing also to their favorable properties as high chemical stability, wide abundance, and low toxicity^16–18^. The photocatalytic effect of Fe hydroxide was reported to exceed that of Fe oxides under visible light irradiation, due to enhanced generation of ^**·**^OH radicals^19^. It has been also shown, that in the nanometer range particles size is an essential factor, determining the bad gap values and absorption in the UV–Visible spectral range^20^. However, results reported on incident photon-to-photocurrent efficiency and photocatalytic activity of Fe oxides towards organic molecules are still controversial and the implied photochemical mechanisms are under debate^17,18^. The large differences between the measured photodegradation quantum yields were attributed principally to the nature of organic molecules^17^. Strongly reducing or complexing molecules, which react with ferric ions even under dark conditions were found to decompose more easily when irradiated in the presence of iron oxide particles. Oppositely, photocatalytic decomposition quantum yields were negligible in case of organic molecules without strong reducing or complexing activities under dark conditions^17^. Nevertheless, there is still an incomplete understanding of the possible mechanisms controlling the implied photocatalytic processes. As compared to other transition metal oxides, such as Ti, W, Zn, or Sn, photocatalytic properties of Fe oxides are much less investigated for degradation of organic molecules either under UV or visible light irradiation conditions and the studies are mainly limited to model compounds as organic dyes and in a much less extent to other type of organic contaminants^18^.

Along the high recombination ability of the photogenerated charge carriers, aggregation of photocatalytic metal oxide nanoparticles is another drawback, photocatalytic activity strongly depending on the effective surface area of the catalysts^21–23^. The addition of highly conducting carbon-based nanomaterials as activated carbon, carbon nanotubes, fullerenes, or graphene-like platelets was found to be a promising way to prevent both nanoparticle aggregation and charge recombination^21–24^. Furthermore, graphene and graphene-like materials were reported to possess also excellent adsorption properties, contributing to the organic molecules’ removal from contaminated water solutions^14,24^.

This study focuses on the investigations of visible light source induced photocatalytic activity of Fe oxide/Fe hydroxide/graphene-like reduced and N doped graphene oxide (N-rGO) hybrid nanocomposite layers for decomposition of CAP molecules. It is worth to mention that according to previous investigations, the photocatalytic degradation efficiency of antibiotic molecules in the presence of Fe oxide/GO compounds was found to exceed the efficiencies of TiO_2_ or ZnO catalysts under UV irradiation conditions^25^. Moreover, ternary heterojunctions as Ce^3+^/Ce^4+^ doped Fe_3_O_4_ nanoparticles anchored onto GO sheets, Fe_3−x_Ce_x_O_4_/GO^26^ or ZnO–ZnFe_2_O_4_ decorated graphene quantum dots^27^ were reported to have significant photocatalytic efficiencies for degradation of antibiotics under visible light irradiation. We used a single-step environmentally friendly laser-based method for the synthesis and deposition of hybrid layers. Most techniques currently used for the synthesis and deposition of composite surface layers are based on multi-step chemical processes which frequently involve surfactants, with the inconvenience of the presence of residual organic impurities on the catalysts surface, affecting their photoactivity. Our synthesis and deposition method, characterized by reduced processing time, high reproducibility, and versatility, do not involve any chemical product. The Fe oxide/Fe hydroxide/N-rGO hybrid nanocomposites are efficient photocatalysts under visible light irradiation, contrarily to pure Fe oxide/Fe hydroxide or N-rGO layers. Nearly 80% of CAP molecules were degraded in the presence of Fe oxide/Fe hydroxide/N-rGO nanocomposite layer, while the photocatalytic decomposition efficiency of pure Fe iron oxide/Fe hydroxide was limited to a few percent under identical experimental conditions.

## Experimental

### Materials and sample preparation method

Fe oxide/Fe hydroxide/N-rGO hybrid, as well as pure Fe oxide/Fe hydroxide and N-rGO reference surface layers were synthesised with the aid of a matrix assisted pulsed laser evaporation (MAPLE) workstation. The equipment is composed of a stainless steel irradiation chamber purchased from Vacuum Projects, Spain, and a pulsed Nd:YAG laser source (λ = 266 nm, τ_FWHM_ = 4 ns, ν = 10 Hz), Brilliant B, from Quantel, France. As base materials we used Fe oxide, 50–100 nm sized nanoparticles (Sigma-Aldrich) and GO platelets (NanoInnova Technologies). The preparation of the composite targets constituted the first step in the synthesis of the hybrid as well as reference layers. To this aim, the base materials were dispersed in distilled water, within the concentration ranges indicated in Table 1. The aqueous dispersions were submitted to stirring and sonication, and subsequently cooled down until solidification in liquid N_2_. The obtained targets were placed in a double wall target holder inside the irradiation chamber. The holder allows the circulation of liquid N_2_ between its walls, maintaining the dispersions frozen during the laser irradiation experiments. The laser fluence incident on the surface of the targets was set at 0.4 J/cm^2^. 6000 laser pulses were applied for the deposition of each layer. (001) SiO_2_ quartz and (111) Si plates with (1 × /1) cm^2^ surface areas were used as collectors, positioned parallel to the target surface. The distance between the target and collector surfaces was fixed at 4 cm. Before the thin film synthesis and deposition experiments, the irradiation chamber was pumped down until a residual pressure of about 10^–3^ Pa. The irradiations were conducted in controlled N_2_ atmosphere, at a pressure of 20 Pa.

**Table 1:** Concentrations values of Fe oxide and GO platelets base materials used for the preparation of the aqueous dispersions:

Samples	Fe oxide nanoparticles wt. %	GO platelets wt.%
FeO6	6	0
GO6	0	6
FeO6/GO4	6	4
FeO6/GO6	6	6
FeO6/GO8	6	8

### Characterization techniques

The morphology of the layers obtained by MAPLE was investigated by scanning electron microscopy (SEM) and extreme high-resolution field-emission scanning electron microscopy (XHR-FE-SEM) with the aid of a TESCAN VEGA and a FEI Magellan 400L microscopes, respectively. The morphology and chemical composition of the layers were studied by high resolution TEM (HRTEM), scanning transmission electron microscopy (STEM), and energy dispersive X-ray spectroscopy (EDX) with the aid of a FEI Tecnai G2 F20 transmission electron microscope. A high annular angled dark field (HAADF) detector was attached to the STEM microscope, allowing Z-contrast elemental composition mapping^28^.

The chemical bonds between the elements were investigated by attenuated total reflection Fourier transform infrared spectroscopy (ATR-FTIR) and X-ray photoelectron spectroscopy (XPS). The ATR-FTIR spectra were registered in the 3800–400 cm^−1^ wavenumber range, with 4 cm^−1^ resolution using a Shimadzu IR Tracer-100 spectrophotometer. The XPS studies were performed with the aid of a SPECS XPS spectrometer, SPECS Surface Nano Analysis GmbH, Germany, using a monochromatic X-ray source (Al Kα line, 1486.74 eV). The measurements were conducted in ultrahigh vacuum (10^–7^ Pa residual pressure). The spectra were analysed with the SDP XPS software, version 7.0; XPS International, Mountain View, Canada.

UV–Visible diffuse reflectance spectra of the composites were registered with the aid of a Shimadzu UV-2600 spectrophotometer. The absorption edge of the samples was estimated using the Kubelka–Munk function^29^, F(R), (Eq. 1):

1 $${\text{F}}\left( {\text{R}} \right) = \left( {1 - {\text{R}}} \right)^{2} /2{\text{R}}$$

where R is the reflectance of the samples. The direct band gap of the samples was estimated from the first derivative of the Kubelka–Munk function^29^, dF(R)/dλ, and the slope of the function [F(R)hυ]^2^^30^. The band gap energies were determined as being situated at the photon energy value where the slope corresponding to the linear part of the [F(R)hυ]^2^ function intersects the photon energy axis.

### Evaluation of photocatalytic decomposition efficiency

The photocatalytic efficiency of the samples under visible light radiation was determined for the degradation of antibiotic CAP molecules (≥ 98% Sigma-Aldrich, IUPAC Name: 2, 2-dichloro-N-[1, 3-dihydroxy-1-(4-24 nitrophenyl) propan-2-yl]acetamide). To this aim aqueous solutions were prepared, containing 4 ppm CAP molecules. Quartz vials filled with 2 ml CAP solutions were used for the photodecomposition tests. The vials were placed in a photoreactor purchased from Luzchem Research Inc, Canada (LZC-ICH2). 16 visible 8 W lamps (Luzchem LZC-420) with 89% of total emission in the 400–700 nm wavelength range and centered at 420 nm wavelength were used as light source. The total reaction time was 390 min. The photocatalytic activity of the samples was also tested by microvolume total organic carbon (µV-TOC) analyses using a Shimadzu TOC L-CPH apparatus. To this aim the total amount of carbon (TC) and the total amount of inorganic carbon (IC) were determined. 50 µL CAP solutions were injected for TC and IC measurements, respectively. The TOC values were calculated from the difference between TC and IC values. The TOC removal rate was calculated from the equation:

2 $$\upeta_{{{\text{TOC}}}} = \left( {{\text{TOC}}_{0} - {\text{TOC}}} \right)*100/{\text{TOC}}_{0}$$

where TOC_0_ and TOC are the values measured before and during the degradation experiments.

Adsorption tests were also performed under dark conditions (in the absence of radiation) until reaching adsorption/desorption equilibrium of CAP molecules on the surface of the catalyst.

The adsorption and photocatalytic efficiencies of the layers were evaluated from the concentration changes in time of the CAP solutions. To this aim, we measured the peak absorbance of CAP molecules at 276 nm wavelength. The absorption spectra of the CAP solutions were registered at regular time intervals during the photocatalytic tests, using a Shimadzu UV-2600 spectrophotometer. The values were correlated with the concentration of the solutions, as defined by the Lambert–Beer law. The adsorption (η_ads_) and photocatalytic decomposition (η_photocat_) efficiencies of the layers were calculated using the formula:

3 $$\upeta_{{{\text{ads}}/{\text{photocat}}}} = \left( {{\text{C}}_{0} - {\text{C}}} \right)*100/{\text{C}}_{0}$$

where C_0_ is the initial CAP concentration of the aqueous solution and C is the concentration value measured at the regular time intervals during the adsorption or photodegradation tests.

The role of the active species in the light induced degradation of CAP molecules was investigated by *in-situ* trapping experiments. Ascorbic acid (C_6_H_8_O_6_, AA), ammonium oxalate ((NH_4_)_2_C_2_O_4_, AO), and tert-butyl alcohol ((CH_3_)_3_COH, TBA) as ^**·**^O_2_^−^, h^+^, and·^**·**^OH scavengers, respectively were added to the CAP test solutions. The concentration of the scavengers in the CAP solutions was in all cases 0.5 mM.

## Results and discussion

### Characterization of surface morphology

**Figure 1:**
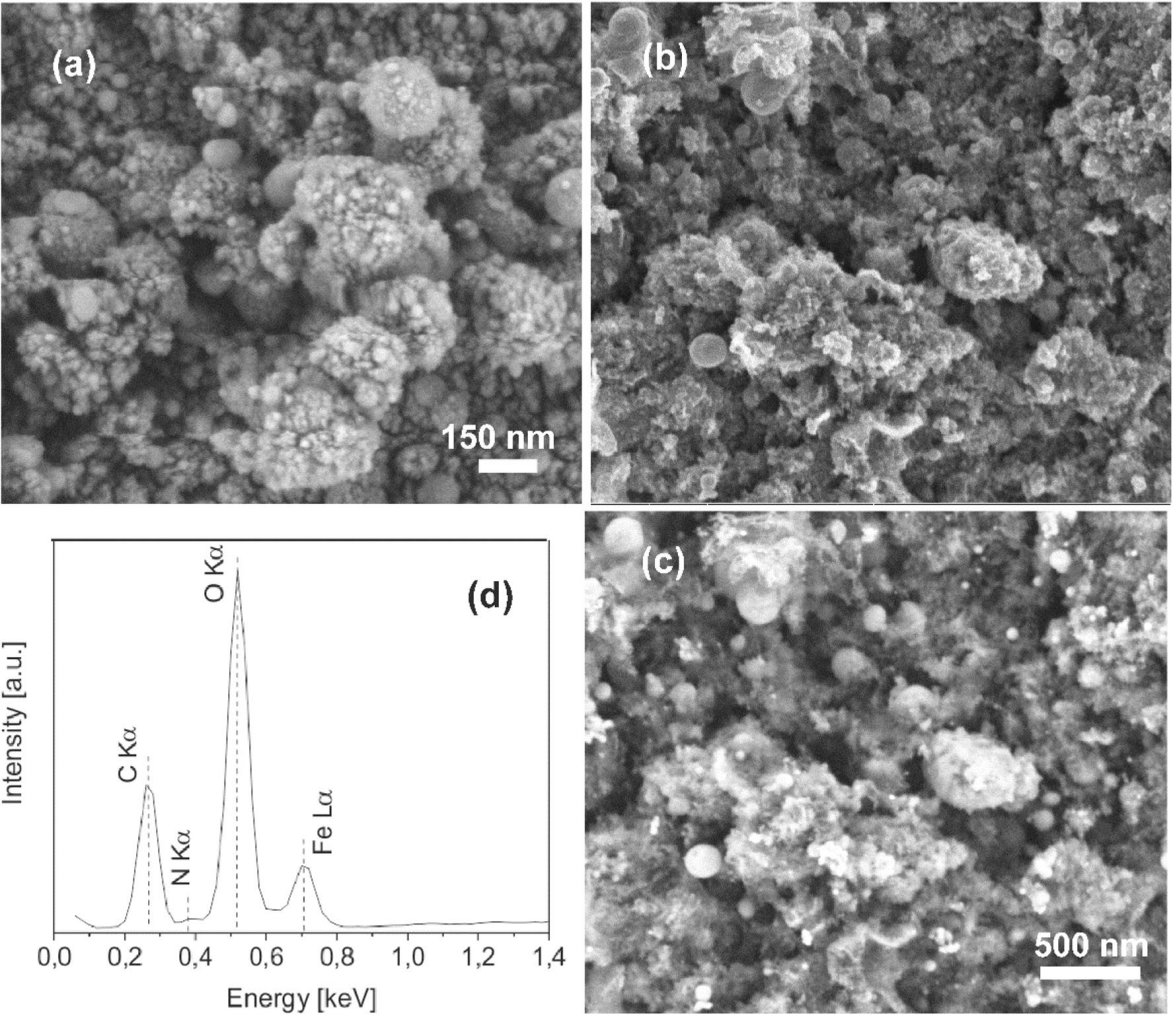
XHR-FE-SEM micrographs of (**a**) pure Fe oxide/Fe hydroxide (FeO6) and (**b,c**) Fe oxide/Fe hydroxide/N-rGO (FeO6/GO6) layers, as well as (**d**) EDX spectrum of the FeO6/GO6 layer; (**b**) SE and (**c**) BSE images of the same surface area.

The XHR-FE-SEM micrographs of pure Fe oxide/Fe hydroxide and hybrid Fe oxide/Fe hydroxide/N-rGO layers are presented in Fig. 1a–c. The surface of the Fe oxide/Fe hydroxide layer (Fig. 1a) is covered by very small, up to tens of nm sized nanoparticles, below the size of the base Fe oxide particles used for the preparation of the MAPLE dispersions (Fig. 1b). The nanoparticles can originate through laser induced melting and dewetting processes or condensation of material evaporated from the target surface.

As compared to the pure Fe oxide/Fe hydroxide layer, the surface of the hybrid Fe oxide/Fe hydroxide/N-rGO layer is more porous (Fig. 1b), constituted by laser transferred GO platelets covered by oxide particles of similar shape and size as in case of the pure Fe oxide layer. From the contrast of the backscattered electrons (BSE) image (Fig. 1c) registered for the same surface area the variation of the composition and the distribution of the Fe containing nanoparticles can be clearly observed. As known, the contras of the BSE image is dependent on the atomic number of the constituent elements, higher atomic number elements, Fe in our composite appearing brighter^31^.

The EDX spectrum of the nanohybrid layer (Fig. 1d) is composed of the O, C, and Fe lines of the constituent compounds, and a less intense N line corresponding to the incorporated nitrogen atoms, the laser irradiations being conducted in N_2_ atmosphere.

### Investigation of chemical composition and chemical bonding states

**Figure 2:**
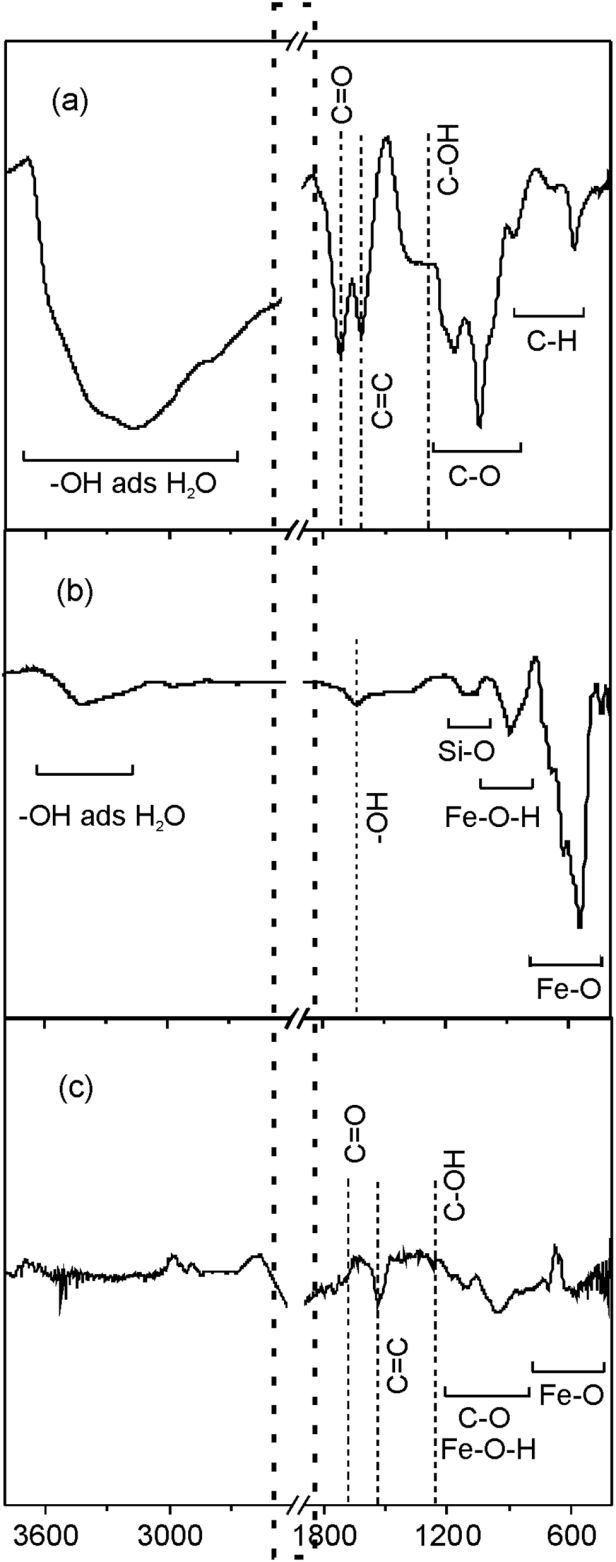
FTIR spectra of (**a**) GO platelets and (**b**) Fe oxide nanoparticles used for the preparation of the MAPLE target dispersions, as well as (**c**) Fe oxide/hydroxide—N-rGO (FeO6/GO6) layer.

The chemical composition and chemical bonding states of the layers were investigated by ATR-FTIR and XPS spectroscopies. The ATR-FTIR spectra of the initial GO platelets and Fe oxide nanoparticles used for the preparation of the target dispersions as well as of the Fe oxide/Fe hydroxide/N-rGO (FeO6/GO6) hybrid layer are presented in Fig. 2.

The marked area between 1966 and 2400 cm^−1^ wavenumbers contains the diamond lines coming from the ATR crystal^32^. The wide band between 3600 and 2600 cm^−1^ in the spectrum of the GO platelets (Fig. 2a) is assigned to the stretching O–H vibration of adsorbed H_2_O molecules and carboxyl acid groups^33–35^. On the other hand, O–H bending vibrations^31,32^ can contribute also to the peak at around 1612 cm^−1^ attributed to C=C stretching vibrations of the graphene aromatic honeycomb structure^33,36,37^.

The band at around 1714 cm^−1^ can be assigned to C=O stretching vibrations of carbonyl and carboxyl groups^33,37^ while the low intensity band at around 1334 cm^−1^ can be attributed to C–OH deformation vibrations of hydroxyl groups^38,39^ of GO platelets. The absorption bands at 1167 and 1038 cm^−1^ can be assigned to asymmetrical and symmetrical C–O–C stretching vibrations, respectively, while the band at 876 cm^−1^ in the ATR-FTIR spectrum could be attributed to C–O–C deformation vibrations of epoxy groups^40^ of GO. Moreover, the weak bands situated in the low wavenumber region were assigned to C-H out of plane bend and wag vibrations^41^.

In the ATR-FTIR spectrum of the Fe oxide nanoparticles (Fig. 2b) the wide band centered at about 3420 cm^−1^ corresponds to O–H stretching vibrations and the peaks at around 1620 and 1085 cm^−1^ to O–H bending vibrations of adsorbed H_2_O molecules^33,34^. The low intensity band situated at around 1085 cm^−1^ could be attributed to Si–O stretching vibrations of the native surface oxide of (111) Si plate used as substrate for the FTIR analysis^42^, while the band centered at around 885 cm^−1^ to Fe–O-H bending vibrations of Fe hydroxide, goethite α-FeOOH, including δ_OH_ in-plane and γ_OH_ out-of-plane bending vibrations^43^. The band centered at 550 cm^−1^ can be assigned to Fe–O stretching vibrations^44^.

The spectrum of the FeO6/GO6 hybrid layer (Fig. 2c) contains bands ascribed to C=C double bonds of graphene aromatic honeycomb structure, single C–O bonds of epoxy functional groups and Fe–O bonds of laser transferred GO platelets and Fe oxide nanoparticles. Additionally, Fe–O–H bending vibrations could superpose with C–O–C deformation vibrations of epoxy groups. The low intensity of the band corresponding to C=O double bonds as well as C–O and C–OH single bonds suggests the reduction of GO platelets during laser processing, leading to the diminishment of the oxygen containing carbonyl, carboxyl, epoxy, and hydroxyl groups.

**Figure 3:**
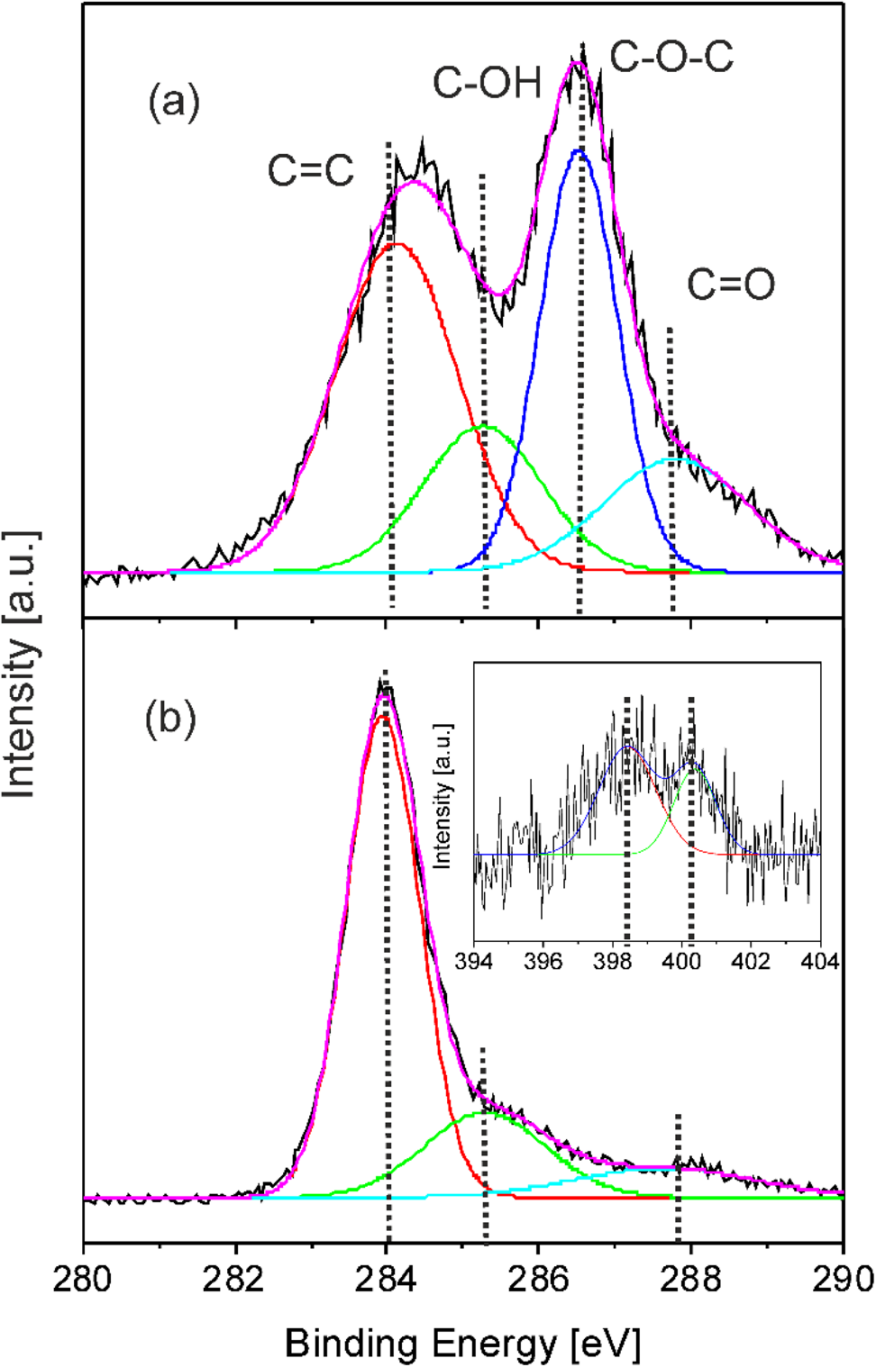
Deconvoluted high resolution C1s XPS spectra of (**a**) GO platelets used for the preparation of the MAPLE target dispersions and (**b**) Fe oxide/Fe hydroxide/N-rGO (FeO6/GO6) layer. The inset corresponds to the deconvoluted N1s spectrum of the FeO6/GO6 layer.

The chemical bonding states between the constituent elements of the layers were investigated also by XPS, recording the C1s, Fe2p, N1s, and O1s high resolution spectra, and were compared with their counterparts belonging to the base materials used for the preparation of the target dispersions. To this aim drop-cast samples were prepared using aqueous dispersions of GO platelets and Fe oxide base materials. The C1s spectra of GO platelets and Fe oxide/Fe hydroxide/N-rGO hybrid layer is presented in Fig. 3.

The spectrum corresponding to the GO platelets (Fig. 3a) was decomposed in four lines, assigned to: (i) C=C double bonds of the graphene aromatic honeycomb structure, which could also contain some contribution from C–C sp^3^ bonds, and lines belonging to oxygen containing functional groups of GO platelets (ii) C–OH single bonds of hydroxyl, (iii) C–O–C single bonds of epoxy, as well as (iv) C=O double bonds of carbonyl and carboxyl groups^45,46^. The intensity of the lines corresponding to C and O single and double bonds diminished significantly, the C–O–C lines being completely reduced in the C1s spectrum of the hybrid layer (Fig. 3b) confirming the results of ATR-FTIR analyses (Fig. 2c) and suggesting the formation of highly reduced, graphene-like material. In the inset of Fig. 3b the N1s spectrum of the Fe oxide/Fe hydroxide/N-rGO layer is presented. The spectrum can be deconvoluted into two components, centered at 398.4 and 400.3 eV binding energies, corresponding to pyridinic, C = N and pyrrolic C-N nitrogen atoms, respectively, confirming the nitrogen doping of rGO platelets^47,48^. In the C1s XPS and ATR-FTIR spectra the contribution of C=N and C–N bonds cannot be clearly distinguished, their binding energies coinciding with those of the carbon–oxygen single and double bonds of the remaining oxygen containing functionalities of rGO platelets^39^.

**Figure 4:**
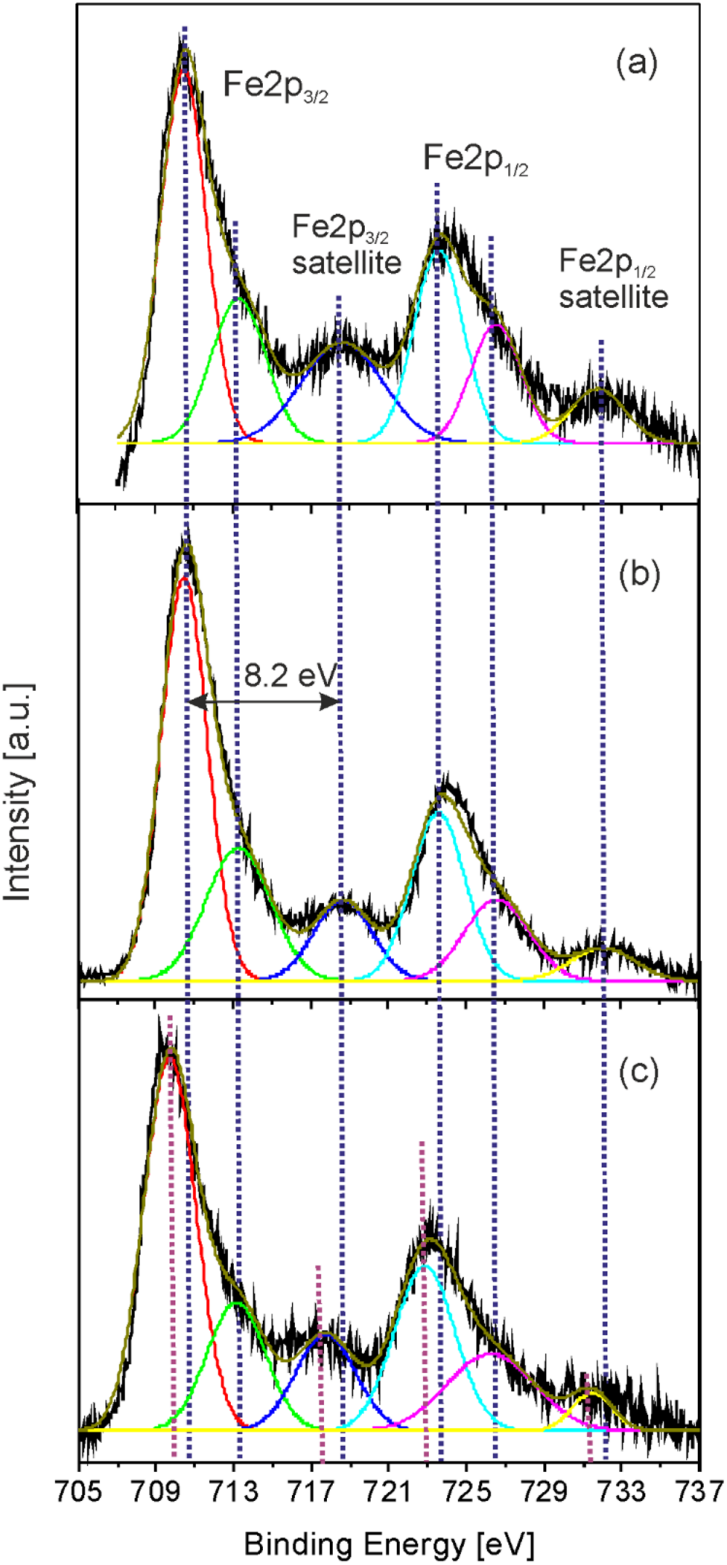
Deconvoluted high resolution Fe2p XPS spectra of (**a**) iron oxide nanoparticles used for the preparation of the MAPLE target dispersions, and (**b**) pure Fe oxide/Fe hydroxide (FeO6) as well as (**c**) Fe oxide/Fe hydroxide/N-rGO (FeO6/GO6) layers.

The high resolution Fe2p XPS spectrum composed of Fe2p3/2 and Fe2p1/2 doublet lines of Fe oxide nanoparticles used for the preparation of the MAPLE targets, as well as pure Fe oxide/Fe hydroxide and Fe oxide/Fe hydroxide/N-rGO layers are shown in Fig. 4. In case of the Fe oxide nanoparticles (Fig. 4a) and pure Fe oxide layer (Fig. 4b) the main Fe2p3/2 and Fe2p1/2 lines are centered at 710.5 and 723.6 eV, accompanied by satellite lines situated at 718.6 and 731.7 eV, respectively^49–51^. Both the position of the Fe2p lines as well as the large energy difference, 8.2 eV between the main Fe2p3/2 and satellite line indicate the presence of Fe^3+^ ions of Fe_2_O_3_ oxide and/or FeOOH hydroxide species, their XPS spectra being very similar^49,50^. The asymmetric shape of the Fe2p3/2 and Fe2p1/2 lines is a characteristic for Fe oxides and hydroxides^49–52^.

The Fe2p spectrum of the composite layer is shifted towards lower binding energy values, with the Fe2p3/2 and Fe2p1/2 lines centered at 709.7 and 722.8 eV, respectively (Fig. 4c). The downshift can be assigned to the presence of Fe^2+^ ions, indicating the formation of Fe_3_O_4_, besides the Fe_2_O_3_ oxide and/or FeOOH hydroxide species^50,52^. The satellite of Fe^2+^ Fe2p3/2 is reported at around 5 eV towards higher binding energies from the main Fe^2+^ line^50,52^. However, the satellite structure of nanosized material is significantly weaker than for the bulk counterparts^53^ and the decrease of the satellite line intensity was directly correlated with the diminishment of the particle size of Fe_3_O_4_ structures^54^. Due to this reasons the unambiguous splitting of the satellite structure centered at 718.6 eV into two components, corresponding to Fe^2+^ and Fe^3+^ main lines remains challenging. Similar reduction of Fe_2_O_3_ in the presence of GO, called graphenothermal reduction, was observed during thermal annealing^55^.

Analogous reduction mechanisms can take place also during laser irradiation of MAPLE target dispersions. The process was related also to the oxidation degree of GO, the reaction pathways being different^55^. At low oxidation degree the reduction process follows a conventional three-stage reaction path, Fe_2_O_3_ → Fe_3_O_4_ → FeO → Fe, whereas GO with higher oxidation degree transforms rapidly Fe_2_O_3_ to Fe_3_O_4_, the processes taking place at 650 °C. On the other hand, the growth of Fe_3_O_4_ or Fe_2_O_3_ layers was reported under UV laser irradiation of pure Fe oxide targets, depending on the nature of the ambient atmosphere N_2_ or O_2_, respectively. The layers were deposited by conventional pulsed laser deposition at pressure values similar to the N_2_ ambient gas pressure used in our experiments^56^. Oppositely to these results, the Fe_3_O_4_ was not detected in the composition of pure Fe oxide layer grown by MAPLE (Fig. 4b).

**Figure 5:**
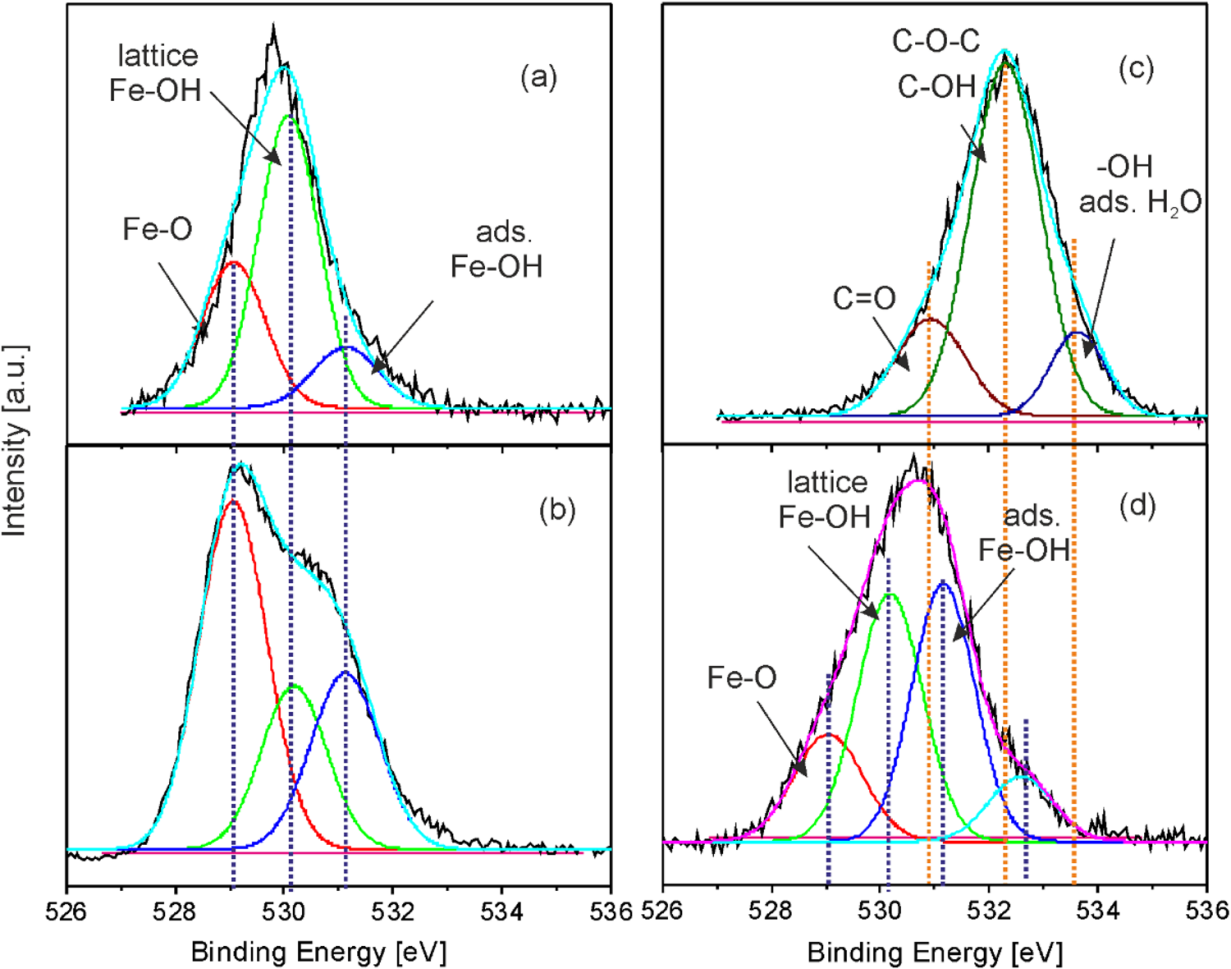
Deconvoluted high resolution O1s XPS spectra of (**a**) Fe oxide nanoparticles and (**c**) GO platelets used for the preparation of the MAPLE target dispersions and (**b**) pure Fe oxide/Fe hydroxide (FeO6) as well as (**d**) Fe oxide/Fe hydroxide/N-rGO (FeO6/GO6) layers.

The high resolution O1s XPS spectra of Fe oxide nanoparticles and GO platelets used for the preparation of the MAPLE target dispersions, as well as pure Fe oxide/Fe hydroxide and Fe oxide/Fe hydroxide/N-rGO hybrid layers are presented in Fig. 5. In the O1s spectra of the Fe oxide nanoparticles (Fig. 5a) and pure Fe oxide/Fe hydroxide layer (Fig. 5b) the lines centered at 529.1, 530.2, and 531.2 eV binding energies can be assigned to lattice oxygen atoms from Fe–O bonds of the Fe_2_O_3_ and FeOOH species, lattice Fe-OH bonds of FeOOH, and Fe-OH bond located on the surface attributed to adsorbed water molecules^57–59^. The peak area ratio of the Fe–O and lattice Fe-OH lines, A_Fe-O_/A_Fe-OH_, changed after laser irradiation from 0.52 to 2.20, attributed to the increase of the number of lattice Fe–O bonds after laser irradiation (Fig. 5a,b). This feature indicates that surface oxidation takes place during laser processing. Indeed, in the case that the surface would contain solely Fe-OH bonds from FeOOH, the ratio A_Fe-O_/A_Fe-OH_ would be approximately one. However, the value below one of the initial nanoparticles suggests the existence of Fe(OH)_2_ besides Fe_2_O_3_ and FeOOH species. The relatively high A_Fe-O_/A_Fe-OH_ value of the laser transferred nanoparticles exceeding one indicates that the main phases include a much higher amount of Fe_2_O_3_ besides FeOOH and Fe(OH)_2_ as compared to the initial nanoparticles. Similar changes in the surface composition were observed upon thermal treatment of sub-micron sized iron oxide particles, leading to oxide formation from lattice hydroxide^52^. According to our numerical simulations assuming photothermal absorption processes, the temperature of oxide nanoparticles in the frozen water matrix reaches hundreds of Kelvins during UV laser irradiation^60^. Thermal transfer to the surrounding water matrix induces a rapid temperature increase, followed by the onset of ablation and transport of the nanoparticles toward the substrate surface by the water vapor^60^.

On the other hand, the relative peak area of adsorbed Fe-OH as compared to the total O1s peak area, A_adsFe-OH_/A_T_, increases from 0.13 for the initial to 0.26 for the laser transferred nanoparticles. Similar results were reported in the literature for metal oxides exposed to water, resulting in the appearance of additional lines at the high energy side of the initial metal-oxide line of the O1s spectrum, attributed to surface metal hydroxides^61,62^.

The O1s spectrum of the GO platelets (Fig. 5c) was deconvoluted in three lines situated at 530.9, 532.3, and 533.6 eV binding energies. The line situated at 530.9 eV was assigned to C=O double bonds of carbonyl and carboxyl, and the line at 532.3 eV to C–O single bonds, C–O–C bonds of epoxy and C–OH bonds of hydroxyl groups^45,46^. The third line centered at 533.6 eV can be assigned to –OH groups of adsorbed water molecules^47–50^. The O1s spectrum of the Fe oxide/Fe hydroxide/N-rGO (FeO6/GO6) layer (Fig. 5d) contains besides the lines assigned to lattice Fe–O bonds, as well as lattice and adsorbed Fe-OH bonds, a fourth line situated at the high energy side. The line can be attributed to the superposition of the contributions of remaining C–OH hydroxyl groups of laser transferred GO platelets and adsorbed H_2_O molecules^45,46,60–62^. The remaining C=O double bonds of carbonyl and carboxyl groups of GO platelets could contribute to the lines assigned to lattice and adsorbed Fe-OH bonds. The C–O–C epoxy groups of GO platelets were reduced during the MAPLE process, as indicated also by the corresponding high resolution C1s (Fig. 3b) and FTIR (Fig. 2c) spectra. The changes of relative concentrations of oxide and hydroxide species of the hybrid layer as compared to those of the initial oxide nanoparticles (Fig. 5b), cannot be explicitly calculated from the A_Fe-O_/A_Fe-OH_ and A_adsFe-OH_/A_T_ peak areas, due to the superposition of the peaks with the remaining carbon–oxygen single and double bonds of GO platelets. The XPS analyses confirm the ATR-FTIR results suggesting the presence of Fe–OH bonds, through the band centered at around 885 cm^−1^ (Fig. 2b,c) attributed to Fe–OH bending vibrations of goethite α-FeOOH. Additionally, Fe–O stretching lattice vibrations of goethite α-FeOOH can contribute also to the band centered at 550 cm^−1^^43,44,63^.

**Figure 6:**
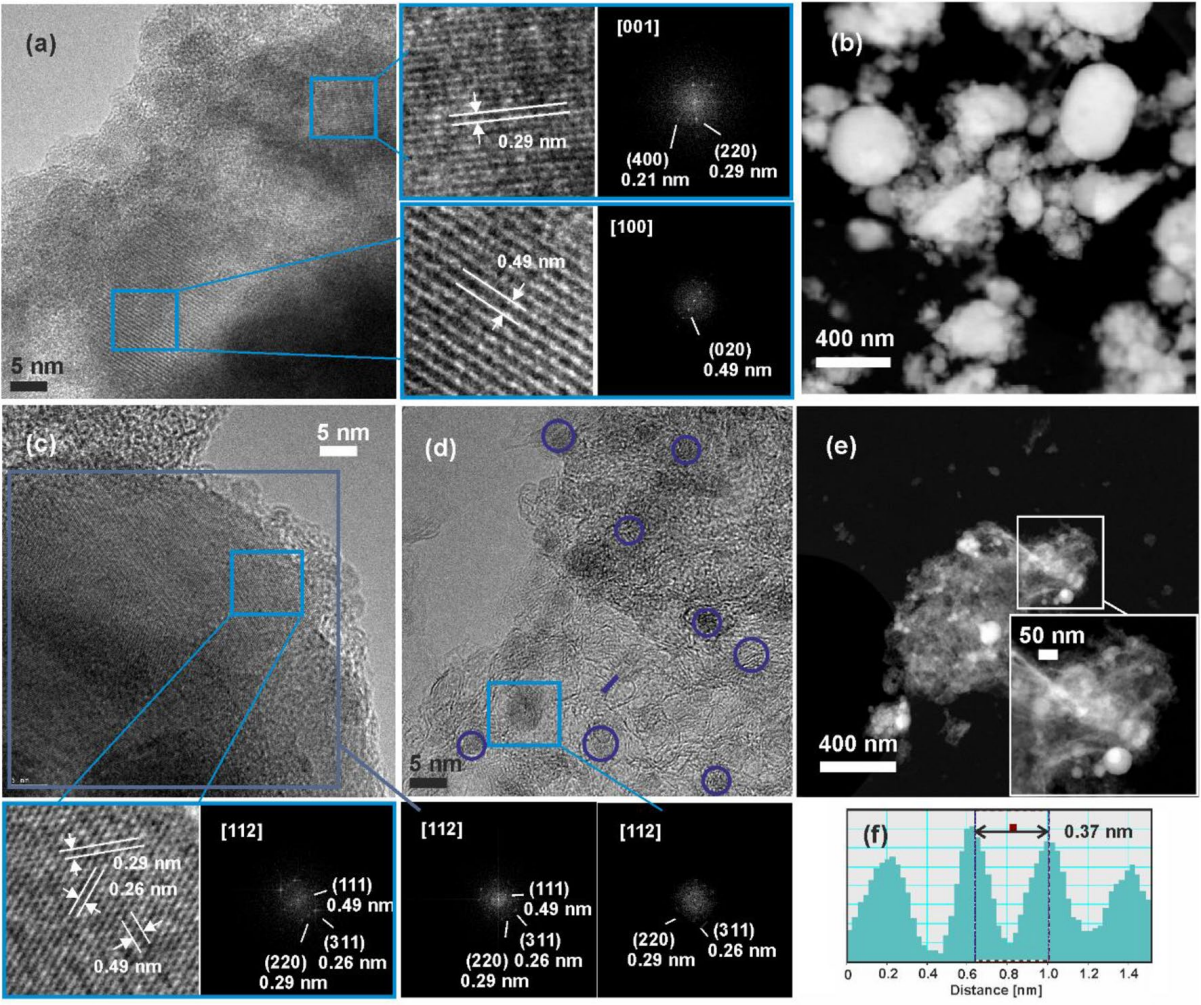
HRTEM micrographs (**a**,**c**,**d**) and corresponding FFT patterns as well as HAADF-STEM micrographs (**b**,**e**) of (**a**,**b**) pure Fe oxide/Fe hydroxide (FeO6) and (**c**–**e**) Fe oxide/Fe hydroxide/N-rGO (FeO6/GO6) layers, and (**f**) line profile along the blue line in (**d**).

STEM and HRTEM analyses were performed in order to further investigate the composition and crystalline structure of the pure Fe oxide and hybrid layers (Fig. 6). The HAADF-STEM images (Fig. 6b,e) confirmed the wide size distribution range of the constituent Fe oxide nanoparticles, in accordance with the results of the XHR-FE-SEM investigations presented in Fig. 1. In case of the nanocomposite layers, the contrast of the HAADF-STEM images allows the identification of metal containing particles, the contrast of the image being proportional to the atomic weight of the constituting elements allowing compositional mapping, larger atomic weight elements appearing brighter in HAADF-STEM images (Fig. 6e). As it can be observed in Fig. 6e, the laser transferred GO platelets are covered by Fe containing particles of spherical shape. The FFT spot patterns corresponding to the marked areas in the HRTEM micrographs (Fig. 6a,c,d) indicate that the pure Fe oxide and nanocomposite layers are formed by single-crystalline nanoparticles. Interplanar distances of 0.29 and 0.49 nm can be measured in the HRTEM micrograph of the pure Fe oxide layer (Fig. 6a). The 0.29 nm interplanar distance could correspond to the (220) crystal planes of cubic or tetragonal phase Fe_2_O_3_ maghemite (JCPDS 00-039-1346 and 04-008-3650), or cubic phase Fe_3_O_4_ magnetite (JCPDS 00-019-0629), observed from the [001] zone axis direction. Due to their similar crystal structure, these phases cannot be unambiguously distinguished, maghemite being associated to Fe^2+^ deficient magnetite^64^. The interplanar distance of 0.49 nm could correspond to the (020) crystal plane of goethite α-FeOOH iron hydroxide nanoparticles (JCPDS 00–029-0713), observed along the [100] zone axis.

The HRTEM images of the hybrid samples confirmed that the laser transferred nanoparticles are highly crystalline (Fig. 6c,d). The measured interplanar distances, both for larger (Fig. 6c), as well as few nm sized particles distributed on the surface of N-rGO platelets (Fig. 6d) 0.49, 0.29, and 0.26 nm correspond to the (111), (220), and (311) interplanar distances of cubic phase Fe_2_O_3_ or Fe_3_O_4_, observed along the [112] direction. Moreover, interplanar distances of around 0.37 nm were measured for the hybrid layer (Fig. 6f), corresponding to the (0002) lattice planes of laser transferred GO platelets, exceeding the value of pure graphite, 0.34 nm [JCPDS card number 75-2078], due to the presence of oxygen and nitrogen containing functional groups^65^.

### Study of photocatalytic properties and photochemical stability of the hybrid photocatalyst under visible light irradiation

**Figure 7:**
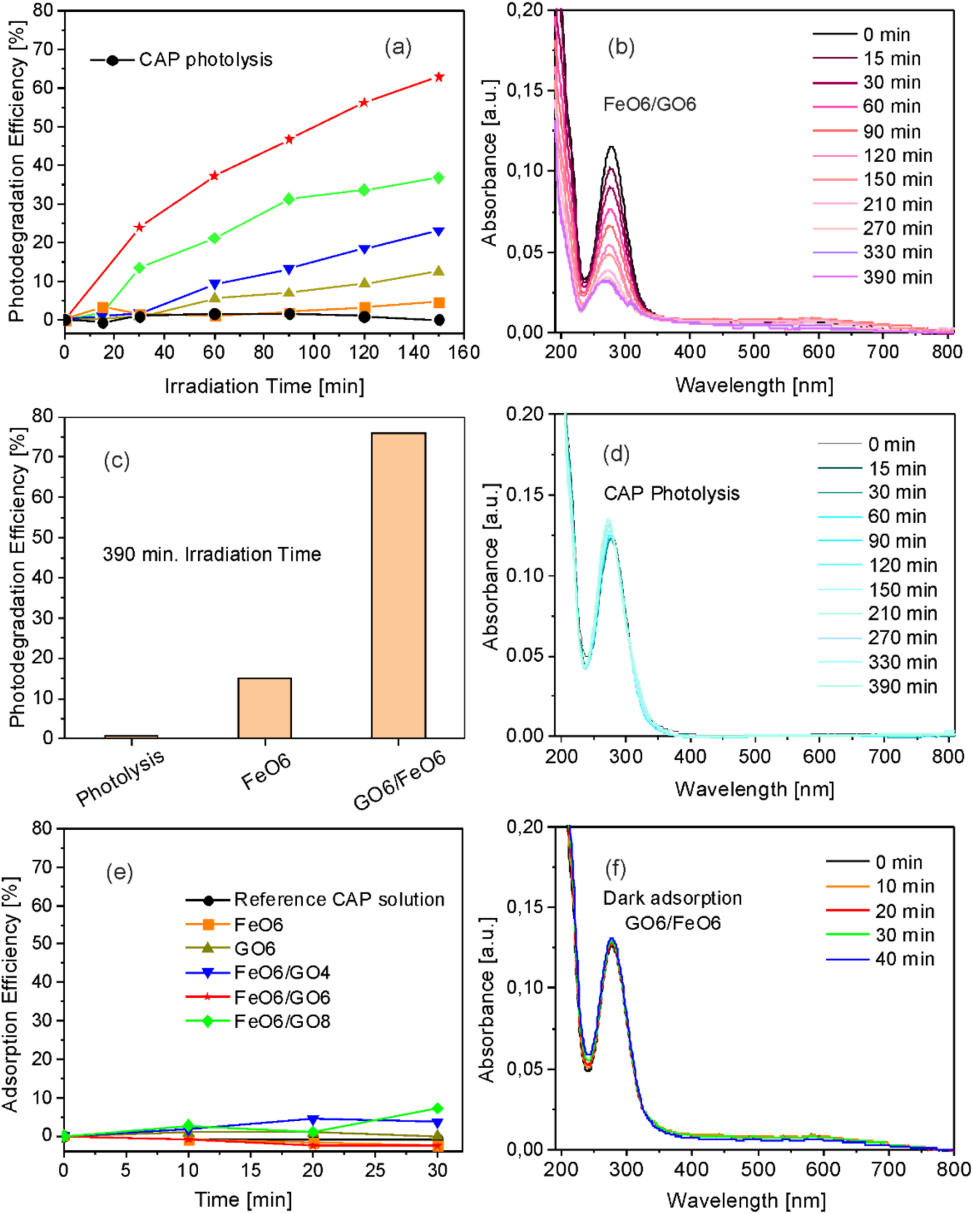
(**a**) Photolysis and photocatalytic decomposition efficiency towards degradation of CAP molecules in aqueous solutions during 150 min. visible light irradiation of FeO6 (filled rectangle), GO6 (filled triangle), FeO6/GO4 (filled inverted triangle), FeO6/GO6 (filled star), and FeO6/GO8 layers (filled diamond), (**b**) UV–Visible spectra of CAP aqueous solutions under visible light irradiation, measured at regular time intervals, photolysis in the absence of catalytic layers, (**c**) photolysis and photocatalytic degradation efficiency towards degradation of CAP in aqueous solutions after 390 min. Visible light irradiation in the presence of FeO6 and FeO6/GO6 layers, (**d**) UV–Visible spectra of CAP aqueous solutions under visible light irradiation, measured at regular time intervals in the presence of FeO6/GO6 layer, (**e**) adsorption efficiency towards CAP in aqueous solutions under dark conditions of FeO6 (filled rectangle), GO6 (filled triangle), FeO6/GO4 (filled inverted triangle), FeO6/GO6 (filled star), and FeO6/GO8 layers (filled diamond), (**f**) UV–Visible spectra of CAP aqueous solutions under dark conditions, measured at regular time intervals, in the presence of the FeO6/GO6 layer.

The results of photocatalytic test of reference pure Fe oxide/Fe hydroxide and N-rGO layers as well as hybrid compounds towards degradation of CAP molecules during 150 min. visible light irradiation and photolysis of CAP molecules in the absence of photocatalysts are presented in Fig. 7. In the absence of photocatalysts the UV–Visible absorbance spectrum of CAP solution remains unchanged, indicating that no photolysis takes place during the test period (Fig. 7d). Moreover, the pure Fe oxide/Fe hydroxide layer (FeO6) has very low degradation efficiency, of the order of a few percent after 150 min. (Fig. 7a), reaching around 15% after 390 min. of irradiation (Fig. 7c), confirming the low photocatalytic decomposition efficiency of Fe oxides reported in the literature for organic molecules which have no reducing or complexing activities under dark conditions^17^. The adsorption efficiencies of both pure Fe oxide/Fe hydroxide and GO layers as well as Fe oxide/Fe hydroxide/N-rGO layers were found to be negligible under dark conditions (Fig. 7e,f).

**Figure 8:**
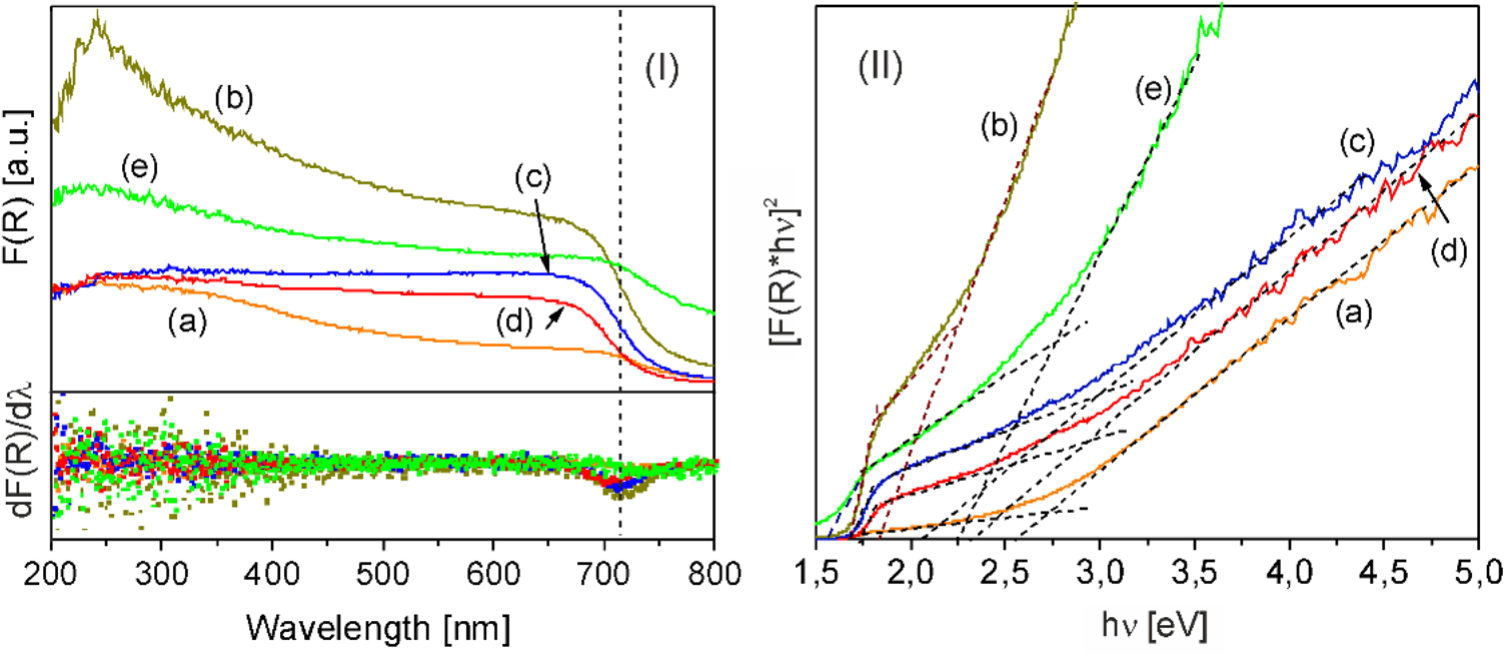
(**I**) (top) Kubelka–Munk functions and (bottom) first derivatives as well as (**II**) [F(R) x hυ]^2^ function versus hυ plots of the (a) Fe oxide/Fe hydroxide (FeO6), (b) RGO (GO6), and Fe oxide/Fe hydroxide/N-rGO (c) FeO6/GO4, (d) FeO6/GO6 (e) FeO6/GO8 layers.

The band gap values of the pure and hybrid layers were evaluated from the first derivative of the Kubelka–Munk function, dF(R)/dλ, and the slope of the function [F(R)hυ]^2^ (Fig. 8). The linear fit was applied for the slope below and above the absorption edge of the composite Fe oxide/Fe hydroxide and Fe oxide/Fe hydroxide/N-rGO layers, the intersection of the two fitting lines giving the band gap energy^66^. For the pure GO6 layer a band gap range was determined, in accordance with reported data for rGO^67^. Similarly, the band gap range was estimated for the rGO containing composite samples. The calculated values, between 1.57 and 2.99 eV, indicate that the layers absorb the whole spectral range, 400–700 nm of the lamps used for the photocatalytic tests (Table 2).

**Table 2:** Band gap values of Fe oxide/Fe hydroxide (FeO6), GO oxide (GO6), and hybrid Fe oxide/Fe hydroxide/N-rGO, FeO6/GO4, FeO6/GO6, and FeO6/GO8 layers calculated from the slope of the [F(R)hυ]^2^ function versus incident photon energy, hυ.

Samples	Band gap [eV]
FeO6	2.67
GO6	1.69–2.24
FeO6/GO4	1.68–2.98
FeO6/GO6	1.73–2.89
FeO6/GO8	1.57–2.79

However, the photocatalytic decomposition efficiency of pure N-rGO layer (GO6) although exceeds the efficiency of pure Fe oxide/Fe hydroxide layer (FeO6), remains lower than the values measured for the hybrid layers, reaching around 23 and 65% for the Fe oxide/Fe hydroxide/N-rGO layers, FeO6/GO4 and FeO6/GO6, respectively after 150 min. visible light irradiation (Fig. 7a). The photodegradation efficiency of the FeO6/GO6 layer calculated from the UV–Visible absorbance spectrum of CAP solution (Fig. 7b) reaches nearly 80% after 390 min. of irradiation (Fig. 7c). However, the TOC removal rate remains about 20% lower as compared to the measured photocatalytic decomposition efficiency values, indicating the formation of a low quantity of smaller molecular weight products, in accordance with previous antibiotics decomposition studies^68^.

Table 3 contains the main parameters of photocatalytic process, nature of photocatalyts, preparation methods, degradation efficiency, as well as irradiation domain and time reported in the literature. As compared to UV light induced photocatalytic processes, achievement of high decomposition rate of CAP molecules under visible light require complex multicomponent materials, which imply multistep synthesis methods and often prolonged irradiation times.

**Table 3:** Photocatalytic process parameters, nature of photocatalyts, preparation methods, degradation efficiency, as well as irradiation domain and time.

Photocatalyst	Synthesis method	CAP degradation efficiency [%]	Radiation	Refs.
Ag/TiO_2_ nanoparticles	chemical reduction	84	UV light, 120 min	^69^
Fe(III)-hydroxysulfate/oxalic acid	bio-synthesis	97.5	UV to IR, 40 min	^70^
TiO_2_ nanoparticles (30% rutile, 70% anatase)	commercial	93.8	UV light, 45 min	^71^
TiO_2_-H_2_O_2_	commercial	99.5	UV light, 180 min	^72^
B, Ni co-doped TiO_2_/g-C_3_N_4_	sol gel, calcination, and heating reflux method	95	Visible light, 180 min	^73^
CdS/WO_3_/FTO photoanode; hemin-graphene cathode—photoelectrocatalysis	layer-by-layer assembly of CdS quantum dots on WO_3_/FTO	80	Visible light, 10 h	^74^
SrFeO_3−*x*_/g-C_3_N_4_	sintering	95	Visible light, 24 h	^75^
CuInS_2_	hydrothermal technique	50	Visible light, 160 min	^76^
St-doped La cobaltate/Cl-doped carbon nitride	Calcination, sol–gel	20	Visible light, 20 min	^77^
Fe oxide/Fe hydroxide/N-rGO layers	MAPLE laser based method	80%	Visible light, 400–700 nm, 16 × 8 W lamps; 1 × 1 cm^2^ surface area; ~ 10 μg; 360 min	Data reported in this work

In the case of the composite layers the initial GO platelets were reduced during the laser irradiation and transfer process, and simultaneously doped with N, leading to the formation of a more conductive, graphene-like material acting as electron traps^78,79^, facilitating the separation of electron hole pairs photogenerated on the surface of Fe oxide/hydroxide species (Eq. 4).

4 $${\text{FeOx}}\left[ {{\text{Fe}}_{{2}} {\text{O}}_{{3}} ,\;{\text{FeOOH}}} \right] + {\text{h}}\upnu \left[ {{\text{visible}}} \right] \to {\text{FeOx}}\left( {{\text{e}}_{{{\text{CB}}}}^{ - } } \right) \, + {\text{ FeOx}}\left( {{\text{h}}_{{{\text{VB}}}}^{ + } } \right)$$

In a conventional system, the charge carriers contribute to the formation of reactive O_2_^−^ superoxide and OH hydroxyl radicals with O_2_ and H_2_O molecules adsorbed on the surface of a semiconductor metal oxide photocatalyst through redox reactions^79^. Nevertheless, reduction of adsorbed O_2_ molecules and formation of O_2_^−^ superoxide anion radicals cannot occur in the case of stoichiometric Fe_2_O_3_ or FeOOH compounds since their CB level is more positive than the reduction potential of O_2_ molecules, − 0.28 eV^80^.

**Figure 9:**
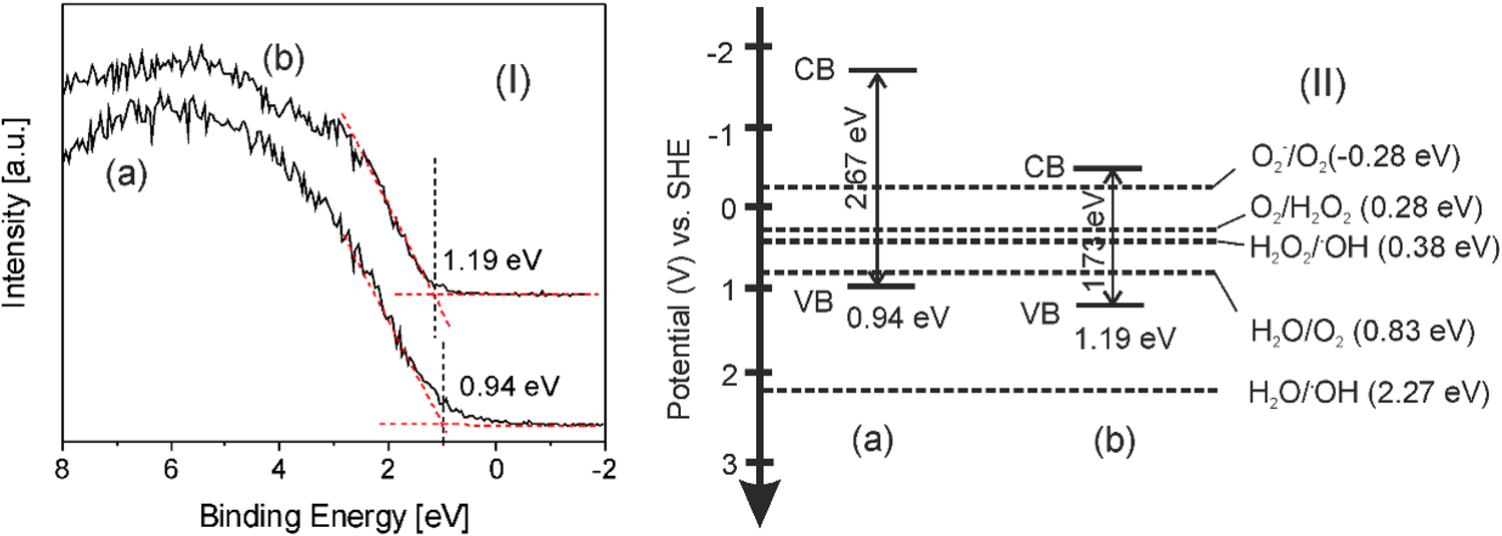
(**I**) VB XPS spectra and (**II**) energy band structures of (a) Fe oxide/Fe hydroxide (FeO6) and (b) Fe oxide/Fe hydroxide/N-rGO (FeO6/GO6) layers vs. SHE, as well as redox potentials at pH 7.

The valence band (VB) maximum levels of Fe oxide/Fe hydroxide and Fe oxide/Fe hydroxide/N-rGO layers determined by VB XPS by linear extrapolation method (Fig. 9I). The VB positions of FeO6 and FeO6GO6 layers are situated at 0.94 and 1.19 eV, respectively. Using the band gap values calculated by UV–Visible spectroscopy (Fig. 8) and the VB position, the conduction band (CB) minimum values of the of FeO6 and FeO6GO6 layers were estimated at − 1.73 and  − 0.54 eV, respectively. The energy band diagram of the samples vs. standard hydrogen electrode (SHE) and redox potentials of reactions relevant with respect to the estimated position of the VB and CB band edges at pH 7^80–82^, are represented in Fig. 9II. Oppositely to pure, stoichiometric Fe_2_O_3_ or FeOOH compounds, the CB level of both the FeO6 and FeO6GO6 layers is more negative than the reduction potential of O_2_ molecules, leading to the generation of O_2_^−^ superoxide radicals from adsorbed O_2_ molecules (Eq. 5):

5 $${\text{O}}_{{2}} + {\text{ FeOx}}\left( {{\text{e}}_{{{\text{CB}}}}^{ - } } \right) \to {\text{O}}_{2}^{ - }$$

Conversely, the oxidation potential of the VB holes is insufficient for the generation of·OH hydroxyl radicals from adsorbed H_2_O molecules, but is more positive than the oxidation potential of H_2_O molecules (Eq. 6):

6 $${\text{2H}}_{{2}} {\text{O}} + {\text{FeOx}}\left( {{\text{h}}_{{{\text{VB}}}}^{ + } } \right) \to {\text{O}}_{{2}} + {\text{ 4H}}^{ + }$$

Moreover, the CB electrons can contribute to the 2e^−^ driven reduction of O_2_ molecules to peroxide (Eq. 7), CB levels being more negative than the reduction potential^80^, leading to the generation of OH radicals (Eq. 8) (Fig. 9II):

7 $${\text{O}}_{{2}} + {\text{2e}}^{ - } + {\text{2H}}^{ + } \to {\text{H}}_{{2}} {\text{O}}_{{2}}$$

8 $${\text{H}}_{{2}} {\text{O}}_{{2}} + {\text{e}}^{ - } \to {\text{OH}} + {\text{OH}}^{ - }$$

**Figure 10:**
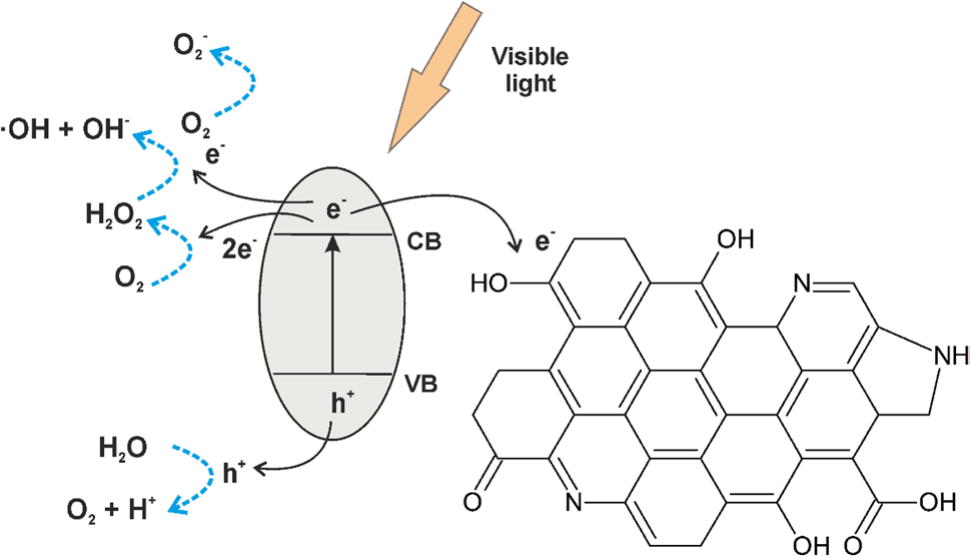
Schematic representation of photon absorption, electron transfer, and generation of reactive species under visible light irradiation of Fe oxide/Fe hydroxide/N-rGO nanocomposite, illustrating also the chemical groups identified by XPS: C–OH hydroxyl, C=O carbonyl, HO–C=O carboxyl, C–N–C pyrrolic-N and C–N=C pyridinic-N.

The nitrogen doped rGO platelets, as indicated by the photocatalytic activity of pure N-rGO (GO6) layer (Fig. 10), besides charge separation, contribute until a certain extent also to the generation of electron–hole pairs (Eq. 9):

9 $${\text{GO}} \to {\text{GO}}\left( {{\text{e}}_{{{\text{CB}}}}^{ - } } \right) + {\text{GO}}\left( {{\text{h}}_{{{\text{VB}}}}^{ + } } \right)$$

Holes and ^**·**^OH radicals are considered to be the primary reactants for oxidative decomposition of organic pollutants^83,84^, mainly through bond breaking of aromatic rings. As opposed to Fe_2_O_3_ or FeOOH, Fe_3_O_4_ present in the composition of the nanohybrid layers cannot participate in the generation of OH hydroxyl radicals, the VB level being situated below the oxidation potential of adsorbed H_2_O molecules^80^. However, the photogenerated electron can be transferred from Fe_2_O_3_ nanoparticles to the half-metallic Fe_3_O_4_, contributing to the reduction of the electron–hole recombination rate owing to its high electrical conductivity^80,85,86^.

**Figure 11:**
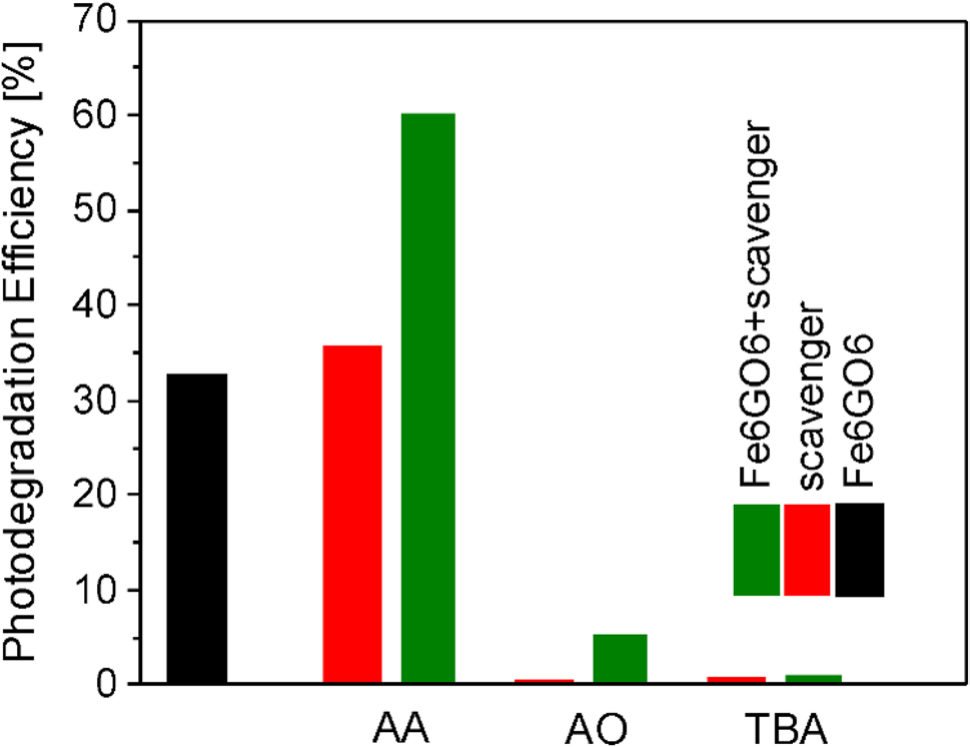
Photodegradation efficiency of the Fe oxide/Fe hydroxide/N-rGO layer (Fe6GO6) layer in the absence and in the presence of AA, AO, and TBA, ^**·**^O_2_^−^, h^+^,·and ^**·**^OH scavengers, respectively, as well as auto-degradation of CAP molecules induced by the addition of scavengers in the absence of photocatalytic layer after 60 min of visible light irradiation.

Control experiments with addition of AA, AO, and TBA scavengers to the test solutions were performed in order to elucidate the role of primary reactants in the degradation of CAP molecules^87^. The degradation efficiency of hybrid Fe oxide/Fe hydroxide/N-rGO layer (Fe6GO6) without and in the presence of scavenger molecules as well as the auto-degradation of CAP molecules induced by the addition of scavengers, in the absence of photocatalytic layer are presented in Fig. 11, after 60 min visible light irradiation.

In the case of AA, the degradation efficiency value is slightly lower than the sum of the contributions from auto-degradation induced by the scavenger and photocatalytic degradation in the presence of the catalytic layer, confirming that ^**·**^O_2_^−^ radicals contribute to the photocatalytic decomposition process. Conversely, the rate of photodegradation was significantly reduced by AO and TBA scavengers, to around 5 and 2%, respectively, conforming the active involvement of h^+^ and ^**·**^OH radicals in the decomposition of CAP molecules.

Additional absorption bands in the UV–Visible spectra were observed frequently during UV light induced photocatalytic decomposition of CAP molecules, indicating by-products formation in the presence of nanohybrid catalyst layers, independently on the nature of semiconductor oxide or carbon-based nanostructures^88–91^. However, no additional absorption bands were observed in the UV–Visible aborption spectra of CAP solutions registered at regular time intervals during the photocatalytic tests (Fig. 7d), confirming the mineralization of CAP molecules.

**Figure 12:**
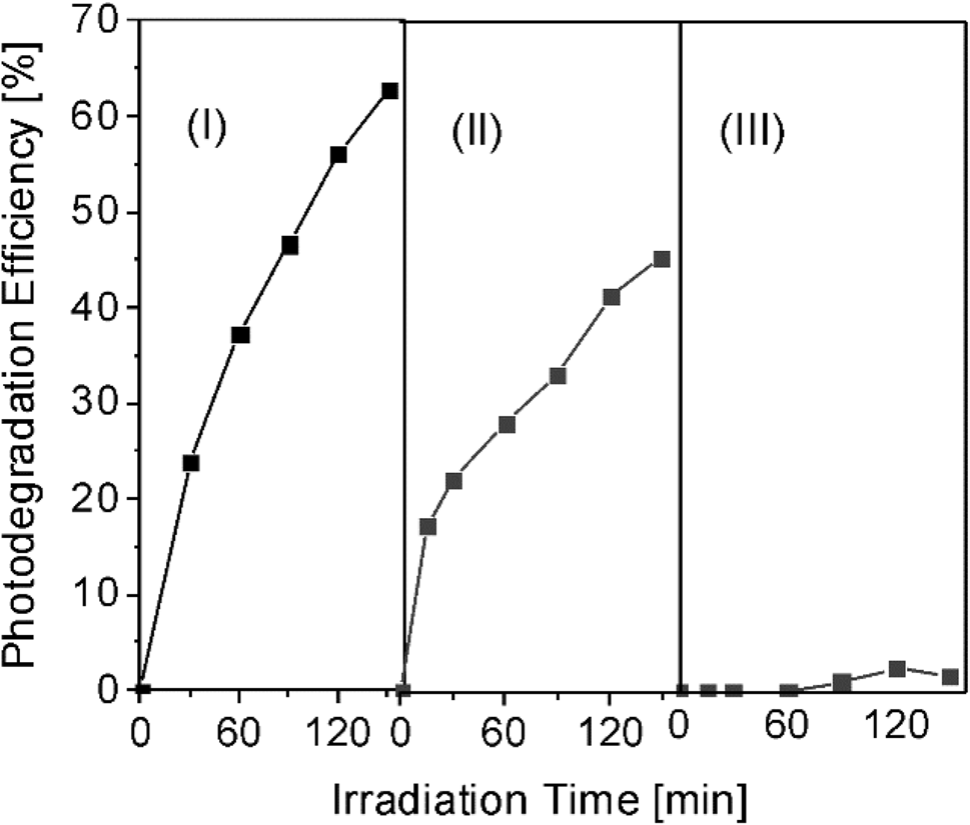
Reusability of the Fe oxide/Fe hydroxide/N-rGO layer (Fe6GO6) layer during three consecutive degradation runs.

The reusability of the composite layers was investigated by consecutive degradation tests (Fig. 12).

The degradation efficiency of Fe oxide/Fe hydroxide/N-rGO, FeO6/GO6 layer decreased from about 65% measured in the first cycle to around 45% during the second degradation run. The layers had no photocatalytic activity during further degradation cycles. The progressive loss of the photocatalytic activity during the successive degradation tests could be due to the adsorption of organic molecules, shielding the catalyst surface from the incident radiation.

The photocatalytic decomposition efficiency of the hybrid Fe oxide/Fe hydroxide/N-rGO layer containing the highest N-rGO concentration (Fe6GO8) was found to be only around 35% after 150 min. of visible light irradiation (Fig. 7a). However, adsorption properties were slightly improved as compared to composites containing lower N-rGO concentrations (Fig. 7e). The decrease of the photocatalytic activity with the increase of the platelets relative concentration was observed also in previous studies^91,92^ and was attributed to the decrease of the active oxide surface area the platelets acting as shields for the oxide particles, hindering the generation of charge carriers at high concentrations.

## Conclusion

Visible-light-responsive photocatalytic nanohybrid layers consisting of Fe oxide and Fe hydroxide nanoparticles as well as graphene–like reduced and nitrogen doped N-rGO platelets were grown for the decomposition of antibiotic molecules in aqueous solutions. A one-step laser-based method was used for the synthesis of the complex nanocomposite layers. The photodegradation efficiency of the layers was investigated as a function of relative concentration of base materials, Fe oxide, GO platelets used for the preparation of the targets submitted to laser irradiation. Adsorption experiments under dark conditions or direct photolysis in the absence of catalytic layers proved that adsorption or photochemical decomposition processes do not contribute significantly to the removal of the antibiotic molecules. Thus, the significant decrease of the antibiotic concentration during the photocatalytic tests can be attributed primarily to light induced photocatalytic decomposition of organic molecules. A possible photodegradation mechanism has also been proposed, based on the comparative study of photocatalytic activities of Fe oxide/Fe hydroxide/N-rGO, pure Fe oxide/Fe hydroxide, and pure N-rGO layers under identical irradiation conditions. The obtained results indicate that the synthesized hybrid layers and the relatively simple synthesis approach could become an eco-friendly and economically feasible alternative treatment technology for the removal of recalcitrant and hazardous pharmaceutical pollutants from water resources.

## Data Availability

All relevant raw data on which the conclusions of the paper rely are presented in the main manuscript file.
